# Metabolomics and Transcriptomics Analyses Explore the Genes Related to the Biosynthesis of Antioxidant Active Ingredient Isoquercetin

**DOI:** 10.3390/foods15020218

**Published:** 2026-01-08

**Authors:** Liyan Cui, Jiaoli Yang, Rui Yuan, Shuting Wang, Zhennan Ma, Defu Wang, Yanbing Niu

**Affiliations:** 1 College of Life Sciences, Shanxi Agricultural University, Jinzhong 030801, China; cuiliyan20191059@163.com (L.C.); yangbao020303@163.com (J.Y.); 17628867627@163.com (R.Y.); 2 College of Grassland Science, Shanxi Agricultural University, Jinzhong 030801, China; 18235488912@163.com (S.W.); m15513998279@163.com (Z.M.)

**Keywords:** *Astragalus membranaceus*, flavonoids, isoquercitrin, metabolitome, transcriptome

## Abstract

*Astragalus membranaceus* is a model of traditional ‘homologous nature of medicine and food’. Its stems and leaves have been proven to have a variety of biological activities. In this study, high-throughput sequencing technology was used to sequence transcriptomics and metabolomics *A. membranaceus* stems and leaves at different growth stages (flowerless stage, flower bud stage, flowering stage, green fruit stage, mature fruit staged, and withering stage), and a regulation analysis was conducted on its differentially expressed genes and differentially accumulated metabolites. The results showed that five hub genes, *PAL*, *CHI*, *AMIE*, *CAD*, and *PRX*, were found to play a central regulatory role in flavonoid biosynthesis. The combined analysis of transcriptomics and metabolomics constructed a flavonoid metabolic regulatory network during the growth and development of *A. membranaceus* stems and leaves. At the same time, based on the significant antioxidant activity of isoquercitrin, three genes that may be related to isoquercitrin biosynthesis were screened, namely *IF7MAT*, *FG3*, and *UGT78D2*. The results of this study provide insights into the biosynthesis and comprehensive development and utilization of flavonoids in *A. membranaceus*.

## 1. Introduction

*Astragalus membranaceus* is a perennial herb of Leguminosae. Its root (Astragali Radix) is a traditional Chinese bulk medicine with immunomodulatory [1], hypoglycemic [2], anti-inflammatory [3], antioxidant [4], and antiviral activities. It first appeared in China’s earliest pharmaceutical monograph “Shen Nong’s Herbal Classic”, and was listed as the first grade in “three-grade classification” [5]. In addition to secondary metabolites, Astragali Radix is also rich in a variety of trace elements and amino acids, so it is widely used in health food [5], such as instant Astragali Radix [6], biscuits [7], tea drinks [8], condiments [9], yogurt [10], and health wines [11]. Its safety has been verified for a long time, and in 2023, it was officially included in the list of ‘homologous nature of medicine and food’ substances by the relevant departments of the Chinese state. As of early 2024, 1558 Astragali Radix health foods products have been approved [12]. In addition, Astragali Radix have been classified as a legal dietary supplement by the U.S. Dietary Supplements Health and Education Act in 1994 [13]. Given the increasing market demand of Radix Astragali and the lack of wild resources, cultivated products have become the main source of commercial Radix Astragali. As of 2020, the planting area of *A. membranaceus* in China has exceeded 1.5 million mu [14]. However, the current production enterprise still has the problem of low resource utilization. About 70% of the by-products (such as stems and leaves) have not been used in high value, and nearly 300,000 tons of waste stems and leaves are produced every year. These stem and leaf resources have not been used reasonably and effectively for a long time [15]. In recent years, the potential utilization value of *A. membranaceus* stems and leaves resources has been gradually explored, such as the development of tea drinks [16], functional foods [17], and other products.

It is worth noting that *A. membranaceus* stems and leaves are also rich in a variety of bioactive components, especially flavonoids, which have high potential for development and utilization. Flavonoids are a kind of yellow pigment derived from 2-phenylchromone, and their main active structure is a hydroxyl substituent. Flavonoids widely distributed in plants have a variety of biological functions, such as regulating plant growth, protecting plants from UV damage, signal transduction in plant–microbe interactions, and as phytoalexins and active oxygen scavengers. At the same time, flavonoids are also a natural health food additive, which can be used as natural antioxidants [18], sweeteners [19], pigments [20], and food preservatives [21]. Flavonoids have become a hot spot in the development and utilization of natural medicines and health products due to their therapeutic and preventive functions in protecting cardiovascular health, anti-oxidation, inhibiting tumors, and regulating immunity [22]. More than 30 kinds of effective bioactive components of flavonoids exist in *A. membranaceus*, including flavonoids, isoflavones, and pterostane [23]. Various bioactive components in *A. membranaceus* have gradually made progress in the fields of beauty and health care. These compounds can improve the immune system, protect against damage due to free radicals, slow down the aging process, prevent oxidation, and fight against bacterial infections [24].

In order to deeply analyze the biosynthesis and regulation mechanism of active ingredients such as flavonoids in *A. membranaceus*, modern omics techniques such as combined analysis of transcriptomics and metabolomics have become key research methods. The combined analysis of transcriptomics and metabolomics is becoming increasingly and widely used in the biosynthesis of plant flavonoids. Xue et al. used transcriptomic and metabolomic techniques to analyze and clarify that the flower color change of *Lonicera japonica* Thunb. may be related to the regulation of chlorophyll and carotene [25]. The accumulation of anthocyanin in *Asparagus officinalis* L. is related to light [26]. Jiang et al. showed that the flesh color of *Actinidia arguta* (Siebold & Zucc.) Planch. ex Miq. is related to the regulation mechanism of flavonoids [27]. The combined analysis of transcriptomics and metabolomics of *Carthamus tinctorius* L. at different flowering stages showed that *4CL*, *DFR*, and *ANR* were up-regulated with the flowering process, while *CHI*, *F3H*, and *FLS* were down-regulated [28].

On the basis of the current development model of the *A. membranaceus* industry, our previous studies have shown that isoquercitrin has remarkable antioxidant activity in *A. membranaceus* stems and leaves [29]. Therefore, in this study, *A. membranaceus* stems and leaves at different growth stages were used as materials, and high-throughput sequencing technology was used to perform transcriptome and metabolomic analysis and joint analysis to clarify the flavonoid biosynthesis regulatory network in the stems and leaves of *A. membranaceus* and screen the key regulatory genes related to isoquercitrin biosynthesis.

## 2. Materials and Methods

### 2.1. Plant Material

*A. membranaceus* stems and leaves were collected from the experimental base (37°25′17′′ N, 112°34′41′′ E) of the College of Life Sciences of Shanxi Agricultural University in Taigu District, Shanxi Province. The two-year plants with the same growth status were collected at six different stages: flowerless stage (FL: plants before flowering), flower bud stage (FB: 50% of the plants entered the budding stage), flowering stage (F: 50% of the plants entered full-bloom stage), green fruit stage (GF: pod formation and initial development), mature fruit stage (MF: pod filling completed), and withering stage (W: leaves began to turn yellow and fall off). The stems and leaves of the whole plant were collected and mixed at six different growth stages. The samples were collected at three biological replicates in each stage, frozen in liquid nitrogen, and stored at −80 °C.

### 2.2. Widely Targeted Metabolomic Analysis and Annotation

About 50 mg of dried sample powder was added to 1200 μL of pre-cooled 70% methanol aqueous solution for metabolite extraction. The mixture was centrifuged at 12,000 rpm for 3 min, and the supernatant was obtained for UPLC-ESI-MS/MS analysis. Electrospray ionization source was used for mass spectrometry analysis, the temperature was 550 °C, and the ion spray voltage was 5500 V/−4500 V (positive/negative ion mode). Ion source gas I (GS I), gas II (GS II), and curtain gas (CUR) were 50, 60, and 25 psi, respectively, and the scanning mode was MRM. Principal component analysis (PCA) was performed by the statistical function prcomp in R (www.r-project.org). The variable importance in projection (VIP) value was extracted from the orthogonal projections to latent structures–discriminant analysis (OPLS-DA) results, and the score map and permutation map were generated with the R package (version 4.5.1) MetaboAnalystR; the model was the best when *p* < 0.05. Preliminary screening of differential metabolites was based on OPLS-DA results [30]. The identified metabolites were annotated using the KEGG compound database (http://www.kegg.jp/kegg/compound/, (accessed on 2 November 2025)), and the annotated metabolites were mapped to the KEGG pathway database (http://www.kegg.jp/kegg/pathway.html, (accessed on 2 November 2025)). The metabolites that were significantly regulated were mapped to certain pathways and then subjected to metabolite set enrichment analysis (MSEA). The significance of these pathways was determined using *p*-values obtained from a hypergeometric test.

### 2.3. Transcriptome Sequencing and Annotation

Total RNA was extracted from *A. membranaceus* stems and leaves by the Trizol method. The Illumina NEBNext^®^ UltraTM RNA Library Prep Kit was used to construct a library with RNA ≥ 1 μg. The library underwent a quality inspection, after which the high-throughput sequencing instrument MGIseq2000 was used. The raw reads were filtered by fastp to obtain high-quality clean reads [31]. The clean reads were spliced by Trinity and de-redundant by corset to obtain the unigene sequence [32,33]. The gene expression was quantified by using bowtie2 in RSEM software [34,35] and evaluated based on the Fragments Per Kilobase of transcript per Million fragments mapped (FPKM) value. FPKM is the ratio of mapped fragments of transcript to the multiplication of total count of mapped fragments (Millions) and length of transcript (kb). Total count of mapped fragments (Millions) is the total number of fragments aligned to the transcripts in 10^6^ units; length of transcript (kb) represents the length of the transcript in 10^3^ bases. TransDecoder (https://github.com/TransDecoder/, (accessed on 2 November 2025)) was used to predict the coding sequence (CDS) of the assembled transcripts. DESeq2 was used for screening differentially expressed genes. DIAMOND BLASTX and HMMER software were used to compare the sequences with the KEGG, NR, Swiss-Prot, GO, KOG, Trembl, and Pfam databases to obtain unigene annotation information [36].

### 2.4. Statistical Analysis

Differentially accumulated metabolites (DAMs) between sample groups were determined by variable importance in projection (VIP > 1), fold change ≥ 2 or fold change ≤ 0.5, and *p* < 0.05 as screening conditions. Differentially expressed genes (DEGs) between sample groups with |log_2_Fold Change| ≥ 1 and false discovery rate (FDR) < 0.05 as screening conditions [37,38]. The key genes related to isoquercitrin were screened with *p*-value ≤ 0.05 as the threshold.

## 3. Results

### 3.1. Overview of the Transcriptome Sequencing

A total of 215.52 Gb clean data were obtained by transcriptome sequencing of 18 samples from six stages of *A. membranaceus* stems and leaves (Supplement Table S1). The clean data of each sample reached 9 Gb, and the percentage of Q30 bases was greater than 91%. After assembly and clustering, 184,121 unigenes were obtained, and a total of 123,960 unigenes were annotated to at least one database. The number of unigenes annotated in the non-redundant (Nr), Translation of EMBL (TrEMBL), Gene Ontology (GO), Kyoto Encyclopedia of Genes and Genomes (KEGG), SwissPort, eukaryotic orthologous groups (KOG), and Pfarm databases was 120,216, 120,116, 103,395, 91,722, 84,236, 73,095, and 71,674, accounting for 67.32%, 65.24%, 56.16%, 49.82%, 45.75%, 39.7%, and 38.93%, respectively (Supplement Figure S1a). A total of 103,395 unigenes in 32 GO terms were annotated to the GO database, accounting for 56.16% (Supplement Figure S1b). Among the biological process (BP, 15), the number of unigenes annotated by cellular process (GO: 0009987) and metabolic process (GO: 0008152) was the largest at 67,492 and 57,667, respectively. Only protein-containing complex (GO: 0032991) and cellular anatomical entity (GO: 0110165) were annotated in cellular component (CC, 2), and the number of unigenes was 14,265 and 79,698, respectively. In molecular function (MF, 15), binding (GO: 0005488) and catalytic activity (GO: 0003824) were the most abundant at 62,469 and 54,197, respectively. A total of 73,095 unigenes were annotated to 25 categories in the KOG database, accounting for 39.7%. The top three categories were general function prediction only; posttranslational modification; and protein turnover, chaperones, and signal transduction mechanisms; the number of annotated unigenes in these categories was 20,934, 7205, and 6800, respectively, and the proportions were 27.90%, 9.86%, and 9.30%, respectively (Supplement Figure S1c). The PCA results of gene expression based on FPKM value showed that the contribution rates of PC1 and PC2 were 18.87% and 15.17%, respectively (Supplement Figure S1d). Correlation analysis between samples revealed that the correlation coefficients between the three repeated samples in each group were greater than 0.8, indicating that the repeatability within the group was good and the correlation was strong (Supplement Figure S1e).

### 3.2. DEGs Were Identified in Six Stages

**Figure 1:**
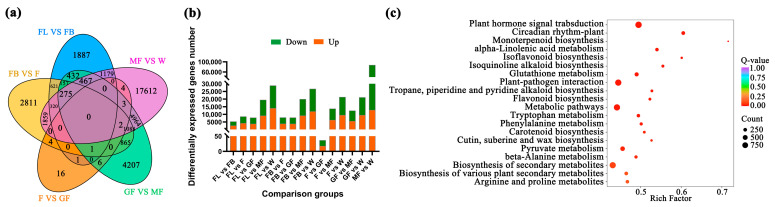
Number of differential genes between different comparison groups. (**a**) DEGs between comparison groups; (**b**) up-/down-regulated DEGs, the green box represents down-regulation, and the orange box represents up-regulation; (**c**) KEGG enrichment analysis of DEGs.

With |log_2_Fold Change| ≥ 1 and false discovery rate (FDR) < 0.05 as the screening conditions, a total of 52,568 DEGs were identified between the sample groups. In the FL vs. FB, FL vs. F, FL vs. GF, FL vs. MF, FL vs. W, FB vs. F, FB vs. GF, FB vs. MF, FB vs. W, F vs. GF, F vs. MF, F vs. W, GF vs. MF, GF vs. W, and MF vs. W comparison groups, the number of up-/down-regulated genes was 2709/2631, 4262/4373, 3888/3995, 9204/10,294, 12,089/14,829, 3943/4060, 3889/4037, 9255/10,749, 12,011/14,733, 18/19, 6365/7368, 9620/11,759, 5648/6819, 9592/11,566, and 12,909/14,864, respectively (Figure 1 and Supplement Table S2). The top 10 KEGG pathways enriched by these DEGs were plant hormone signal transduction, circadian rhythm–plant, monoterpenoid biosynthesis, alpha-Linolenic acid metabolism, isoflavonoid biosynthesis, isoquinoline alkaloid biosynthesis, glutathione metabolism, plant–pathogen interaction, tropane, piperidine and pyridine alkaloid biosynthesis, and flavonoid biosynthesis (Figure 1c).

### 3.3. DEGs Analysis of Flavonoid Synthesis in the Stems and Leaves

**Figure 2:**
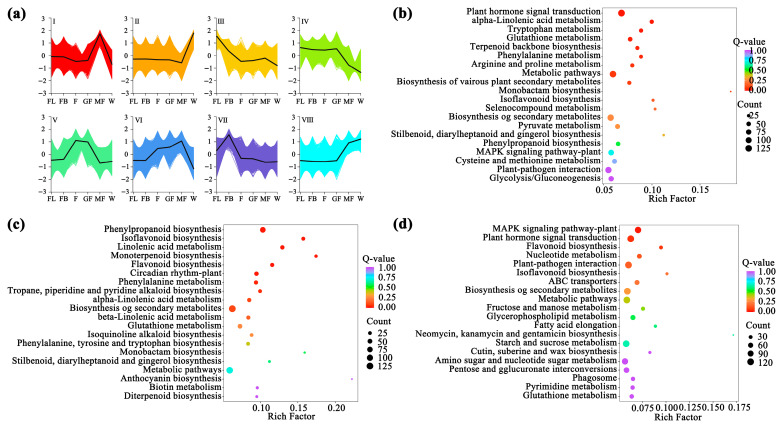
Functional enrichment analysis of flavonoid synthesis DEGs. (**a**) Expression patterns of the eight clusters correspond to the hierarchical cluster result. Eight main clusters are presented as Cluster I–Cluster VIII. Gene expression values are normalized to log10 (FPKM); (**b**) TOP20 KEGG functional enrichment analysis of Cluster-IV DEGs; (**c**) TOP20 KEGG functional enrichment analysis of Cluster-V DEGs; (**d**) TOP20 KEGG functional enrichment analysis of Cluster-VII DEGs.

Trend analysis of DEGs in *A. membranaceus* stems and leaves at six stages revealed eight gene expression patterns, namely Cluster I-VIII (Figure 2a). KEGG functional enrichment analysis was performed on the genes in the eight trends. With Padj ≤ 0.05 as the threshold, the pathway that meets this condition was the pathway that was significantly enriched in the above trend. The results showed that there were three significant enrichment trends (Cluster-IV, Cluster-V, and Cluster-VII) related to flavonoid biosynthesis in different developmental stages of *A. membranaceus* stems and leaves. Among them, the pathway involved in Cluster-IV was phenylalanine metabolism (Figure 2b); the pathways involved in Cluster-V were phenylalanine biosynthesis, isoflavone biosynthesis, flavonoid biosynthesis, and phenylalanine metabolism (Figure 2c); the pathway involved in Cluster-VII was flavonoid biosynthesis (Figure 2d). There were 36 genes annotated to be involved in flavonoid metabolism in the pathway, which regulated the synthesis of related enzymes in the process of flavonoid metabolism in *A. membranaceus* stems and leaves (Supplement Table S3).

### 3.4. Weighted Gene Co-Expression Network (WGCNA) Analysis of Unigenes

**Figure 3:**
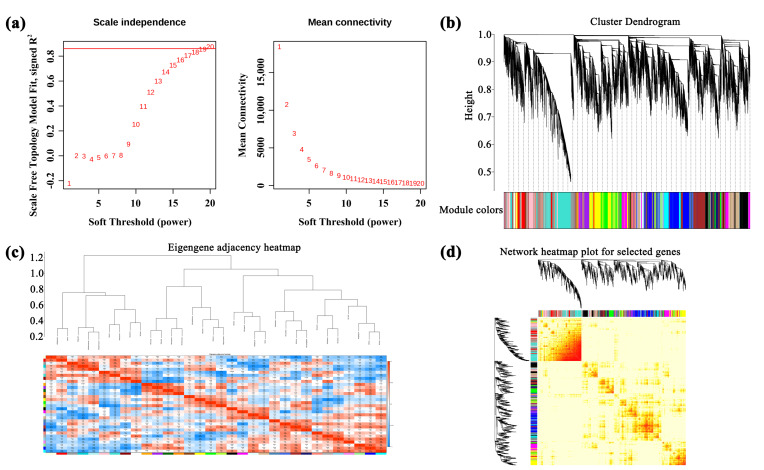
WGCNA analysis. (**a**) Soft threshold selection; (**b**) module hierarchical clustering tree; (**c**) the gene co-expression module of hickory nut; (**d**) cluster analysis of module gene correlation. The position of the red line is represented as a soft threshold.

A total of 184,121 genes were identified in *A. membranaceus* stems and leaves. After removing the genes with low and unstable expression by R language, 36,482 genes were obtained and constructed by weighted gene co-expression network analysis (WGCNA). The square threshold of the correlation coefficient between genes was set to 0.85; that is, R2 > 0.85, the soft threshold (β, power Estimate) was calculated, and β = 19 was selected as the soft threshold (Figure 3a). The clustering tree was constructed according to the correlation of gene expression levels, and the modules were divided by the number of module genes ≥ 50, and the merging threshold of the modules was set to 0.25, resulting in 32 gene modules (Figure 3b). The number of genes in each module was shown in Supplement Table S4. Perform inter module correlation analysis on 32 modules based on their eigengene, and draw a heatmap (Figure 3c). The darker the color of the square (red or blue), the stronger the correlation. Subsequently, the H-clust method was used to cluster the module genes (Figure 3d), where each tree represents a module, and each branch represents a gene, and the darker the color of each point, the stronger the connectivity between the two genes corresponding to the rows and columns.

**Figure 4:**
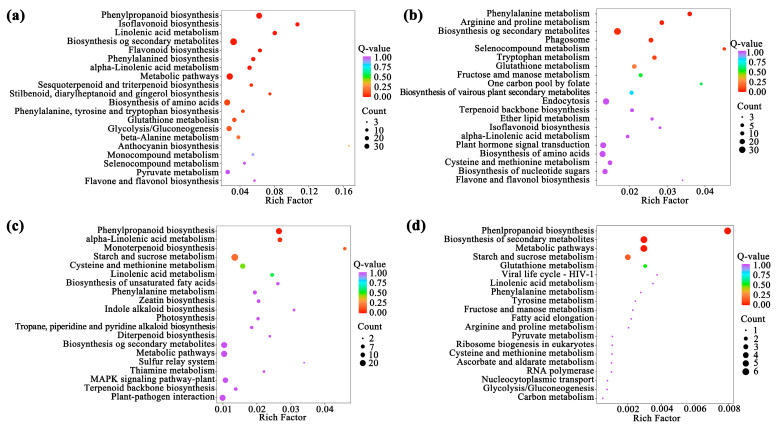
TOP20 KEGG functional enrichment analysis of module genes. (**a**) black module; (**b**) cyan module; (**c**) midnightblue module; (**d**) steelblue module.

KEGG functional enrichment analysis of the above 32 module genes showed that the modules related to flavonoid biosynthesis process during the development of *A. membranaceus* stems and leaves were mainly black, cyan, midnightblue, and steelblue modules. Among them, the pathways related to flavonoid biosynthesis in the black module were phenylpropanoid biosynthesis, isoflavone biosynthesis, flavonoid biosynthesis and phenylalanine metabolism; the cyan module was phenylalanine metabolism; the midnightblue and steelblue modules were phenylpropanoid biosynthesis (Figure 4). Supplement Table S5 listed the genes involved in the flavonoid biosynthesis pathway in four modules.

**Figure 5:**
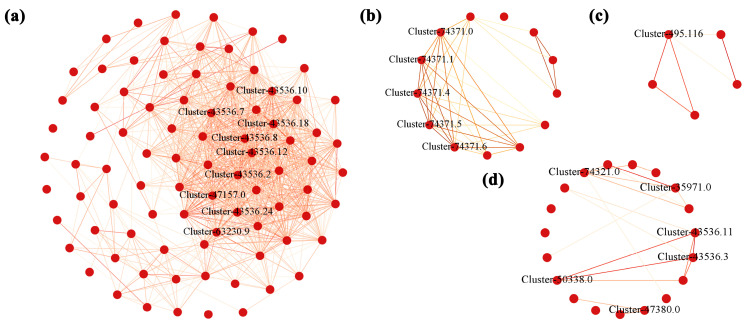
Gene network regulation relationships in four flavonoid biosynthetic modules. (**a**) black; (**b**) cyan; (**c**) steelblue; (**d**) midnightblue. The red underline was marked as the hub gene.

The genes enriched in the flavonoid biosynthesis pathway in the modules of black, cyan, midnightblue, and steelblue were plotted to draw a network regulation map, and the hub gene of flavonoid biosynthesis in the development of *A. membranaceus* stems and leaves was established. The weight value of each gene was determined by the connectivity of the gene, and the genes with high connectivity may play a pivotal role in the module. Therefore, the top 10% of the average connectivity of a single gene in a significant module was used as hub genes. The results showed that the hub genes closely related to flavonoid biosynthesis in the black module were *PAL* (phenylalanine ammonia-lyase: Cluster-43536.18, Cluster-43536.2, Cluster-43536.12, Cluster-43536.10, Cluster-43536.7, Cluster-43536.8, Cluster-43536.24, Cluster-47157.0), and *CHI* (chalcone isomerase: Cluster-63230.9). In the cyan module, it was *AMIE* (amidase: Cluster-74371.0, Cluster-74371.1, Cluster-74371.4, Cluster-74371.5, Cluster-74371.6). In the midnightblue module, there were *CAD* (cinnamyl alcohol dehydrogenase: Cluster-74321.0, Cluster-35971.0), *PAL* (Cluster-43536.11, Cluster-43536.3, Cluster-50338.0), and *PRX* (peroxidase: Cluster-47380.0). In the steelblue module, it was *PRX* (Cluster-495.116) (Figure 5). It can be seen that *PAL*, *CHI*, *AMIE*, *CAD*, and *PRX* play a core regulatory role in the flavonoid biosynthesis and are the hub genes of flavonoid biosynthesis during the development of *A. membranaceus* stems and leaves.

### 3.5. Overview of Sequencing Data and Differential Metabolite Analysis

The mass spectrometry data were processed by Analyst 1.6.1 software. The combined sample’s total ion current (TIC) of the mixed sample and the detection of multiple reaction monitoring (MRM) metabolites’ multiple peaks indicated that the signal for sample analysis signal was robust, the peak capacity was high, and the retention time reproducibility was good (Supplement Figure S2a–d). The integral correction results of quantitative analysis of random metabolites ensured the accuracy of qualitative and quantitative analyses (Supplement Figure S2e–f). The coefficient of variation (CV) and the empirical cumulative distribution function (ECDF) were used to analyze the data distribution. The metabolites with CV values less than 0.3 in quality control (QC) samples accounted for 95.66% of the total metabolites, indicating that the sequencing data were very stable (Supplement Figure S3a). Hierarchical cluster analysis (HCA) of different sample metabolites showed that the repeatability of samples in six stages was good, and the expression trend of metabolites was basically the same (Supplement Figure S3b). The results of sample correlation analysis are shown in Supplement Figure S3c. All three repeated samples in each group had correlation coefficients more than 0.8, indicating excellent repeatability within the group and a strong correlation. The results of principal component analysis (PCA) are shown in Supplement Figure S3d. PC1 and PC2 accounted for 26.44% and 15.8%, respectively. The distinct separation of the six stages indicated significant differences in the accumulation and expression patterns of metabolites throughout these stages. A total of 944 metabolites were identified in *A. membranaceus* stems and leaves (Supplement Figure S3e), of which flavonoids were the most abundant, accounting for 39.51%. The composition of the metabolites in the substance was as follows: phenolic acids (16.74%), terpenoids (15.68%), alkaloids (8.47%), lignans and coumarins (6.14%), quinones (2.44%), tannins (0.74%), and other metabolites (10.28%).

### 3.6. Differential Metabolites of Flavonoids Were Identified in Six Stages

**Figure 6:**
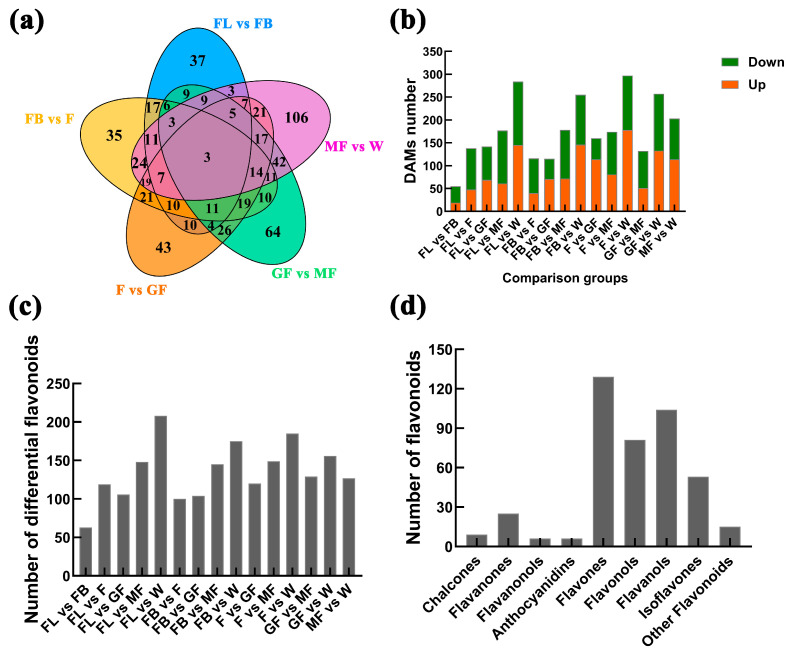
Number of DAMs between different comparison groups. (**a**) DAMs between comparison groups; (**b**) up-/down-regulated DAMs, the green box represents down, the orange box represents up; (**c**) number of differentially accumulated flavonoids; (**d**) classification of differential accumulation flavonoids.

The OPLS-DA analysis of *A. membranaceus* stems and leaves in six stages showed that the metabolites were obviously separated. The prediction parameter Q2 of each comparison group in the model verification is greater than 0.5, indicating that the model was effective (Supplement Figures S4 and S5). DAMs between sample groups were determined by variable importance in projection (VIP > 1), fold change ≥ 2 or fold change ≤ 0.5, and *p* < 0.05 as screening conditions. A total of 770 DAMs were identified in 15 comparison groups (Figure 6a). The up-/down-regulated differential metabolites were 19/36 (FL vs. FB), 48/90 (FL vs. F), 69/73 (FL vs. GF), 61/116 (FL vs. MF), 145/139 (FL vs. W), 40/76 (FB vs. F), 71/44 (FB vs. GF), 72/106 (FB vs. MF), 146/109 (FB vs. W), 114/46 (F vs. GF), 81/93 (F vs. MF), 178/119 (F vs. W), 51/81 (GF vs. MF), 133/124 (GF vs. W), and 114/89 (MF vs. W), as shown in Figure 6b. To clarify the flavonoid biosynthesis regulatory network, we focused on DAMs enriched in the flavonoid biosynthesis pathway. A total of 330 differentially accumulated flavonoids were identified in 15 comparison groups, namely, 63 (FL vs. FB), 119 (FL vs. F), 106 (FL vs. GF), 148 (FL vs. MF), 208 (FL vs. W), 100 (FB vs. F), 104 (FB vs. GF), 145 (FB vs. MF), 175 (FB vs. W), 120 (F vs. GF), 149 (F vs. MF), 185 (F vs. W), 129 (GF vs. MF), 156 (GF vs. W), and 127 (MF vs. W), as shown in Figure 6c and Supplement Table S6. The 330 differentially accumulated flavonoids were divided into nine categories of chalcones (9), flavanones (25), flavanonols (6), anthocyanidins (6), flavones (129), flavonols (81), flavanols (6), isoflavones (53), and other flavonoids (15), as shown in Figure 6d.

### 3.7. Analysis of Flavonoid Biosynthesis Pathway

**Figure 7:**
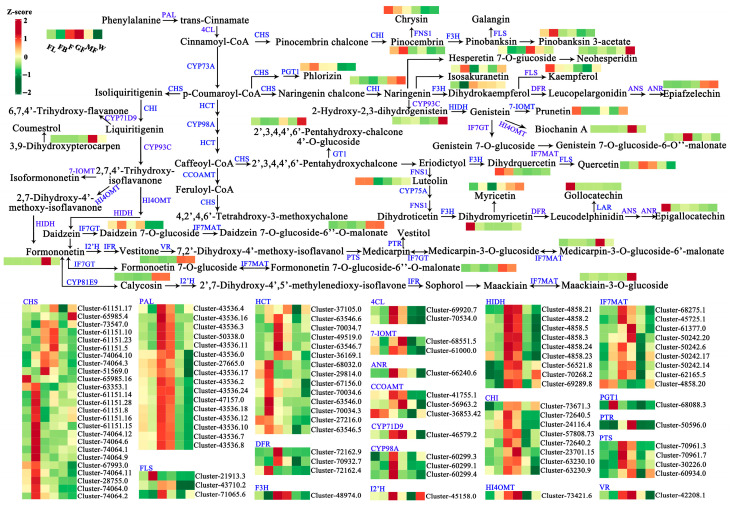
The schematic diagram of flavonoid biosynthesis pathway in *A. membranaceus* stems and leaves. The six columns for each gene and metabolite represent the expression level at FL, FB, F, GF, MF, and W stage, respectively. Red represents the high expression level; green represents the low expression level. *PAL*: phenylalanine ammonia-lyase; *4CL*: 4-coumarate--CoA ligase; *CHS*: chalcone synthase; *CHI*: chalcone isomerase; *FNS1*: flavone synthase I; *F3H*: naringenin 3-dioxygenase; *FLS*: flavonol synthase; *CYP73A*: trans-cinnamate 4-monooxygenase; *PGT1*: phlorizin synthase; *DFR*: bifunctional dihydroflavonol 4-reductase/flavanone 4-reductase; *ANS*: anthocyanidin synthase; *ANR*: anthocyanidin reductase; *HCT*: shikimate O-hydroxycinnamoyltransferase; *CYP98A*: 5-O-(4-coumaroyl)-D-quinate 3′-monooxygenase; *GT1*: chalcone 4′-O-glucosyltransferase; *CYP75A*: flavonoid 3′,5′-hydroxylase; *LAR*: leucoanthocyanidin reductase; *CCOAMT*: caffeoyl-CoA O-methyltransferase; *CYP93C*: 2-hydroxyisoflavanone synthase; *HIDH*: 2-hydroxyisoflavanone dehydratase; *7-IOMT*: isoflavone-7-O-methyltransferase; *HI4OMT*: 2,7,4′-trihydroxyisoflavanone 4′-O-methyltransferase/isoflavone 4′-O-methyltransferase; *IF7GT*: isoflavone 7-O-glucosyltransferase; *IF7MAT*: isoflavone 7-O-glucoside-6′′-O-malonyltransferase; *CYP71D9*: flavonoid 6-hydroxylase; *I2′H*: isoflavone/4′-methoxyisoflavone 2′-hydroxylase; *IFR*: 2′-hydroxyisoflavone reductase; *VR*: vestitone reductase; *PTS*: pterocarpan synthase; *PTR*: pterocarpan reductase; CYP81E9: isoflavone 3′-hydroxylase.

In order to further explore the relationship between DEGs and DEMs involved in flavonoid biosynthesis, this study systematically constructed the flavonoid biosynthesis pathway during the development of *A. membranaceus* stems and leaves (Figure 7). First, *PAL* was used as a catalytic enzyme to catalyze the conversion of Phenylalanine to trans-Cinnamate, followed by the conversion to Coumaroyl-CoA under the catalysis of *4CL*, and then Coumaroyl-CoA opened two pathways (1–2). (1) Coumaroyl-CoA was converted to Pinocembrin (the largest accumulation in FB stage) under the action of *CHS* and *CHI*, and then Pinocembrin opened two pathways (1.1–1.2). (1.1) Pinocembrin was catalyzed by *FNS1* to convert into Chrysin (the largest accumulation in FL stage). (1.2) Pinocembrin was catalyzed by *F3H* to Pinobanksin 3-acetate (the largest accumulation in W stage). (2) Coumaroyl-CoA was converted to p-Coumaroyl-CoA catalyzed by *CYP73A*, followed by four pathways (2.1–2.4). (2.1) p-Coumaroyl-CoA was converted to Phlorizin (the largest accumulation in FB stage) under the catalysis of *CHS* and *PGT1*. (2.2) p-Coumaroyl-CoA was converted to Naringenin (the largest accumulation in W stage) under the action of *CHS* and *CHI*, and four pathways were opened at the same time (2.2.1–2.2.4). (2.2.1) Naringenin was successively transformed into Hesperetin 7-O-giucoside (the largest accumulation in GF stage) and Neohesperidin (the largest accumulation in W stage). (2.2.2) Naringenin converted to Isosakuranetin (the largest accumulation in F stage). (2.2.3) Naringenin was transformed into Dihydrokaempferol (the largest accumulation in F stage) under the action of *F3H*, and two pathways were opened at the same time (2.2.3.1–2.2.3.2). (2.2.3.1) Dihydrokaempferol was converted to Kaempferol (the largest accumulation in FL stage) under the catalysis of *FLS*. (2.2.3.2) Dihydrokaempferol was converted to Epiafzelechin (the largest accumulation in W stage) under the action of *DFR*, *ANS*, and *ANR*. (2.2.4) Naringenin was converted to 2-Hydroxy-2,3-dihydropistein (the largest accumulation in W stage) under the catalysis of *CYP93C*, and then converted to Genistein under the catalysis of *HIDH*, and three pathways were opened (2.2.4.1–2.2.4.3). (2.2.4.1) Genistein was converted to Prunetin (the largest accumulation in FL stage) under the catalysis of *7-IOMT*. (2.2.4.2) Genistein was converted to Biochanin A (the largest accumulation in MF stage) under the catalysis of *HI4OMT*. (2.2.4.3) Genistein was converted to Genistein 7-O-glucoside-6-O’’-malonate (the largest accumulation in F stage) by Genistein 7-O-glucoside catalyzed by *IF7GT*. (2.3) p-Coumaroyl-CoA was converted to Caffeoyl-CoA under the catalysis of *HCT* and *CYP98A*, opening two pathways (2.3.1–2.3.2). (2.3.1) Caffeoyl-CoA was converted to 2′,3,4,4′,6′-Pentahydroxy-chalcone under the catalysis of *CHS*, and two pathways were simultaneously opened (2.3.1.1–2.3.1.2). (2.3.1.1) 2′,3,4,4′,6′-Pentahydroxy-chalcone was converted to 2′,3,4,4′,6′-Pentahydroxy-chalcone 4′-O-glucoside (the largest accumulation in W stage) under the action of *GT1*. (2.3.1.2) 2′,3,4,4′,6′-Pentahydroxy-chalcone converted to Eriodictyol, opening two pathways (2.3.1.2.1–2.3.1.2.2). (2.3.1.2.1) Eriodictyol was converted to Dihydrquercetin (the largest accumulation in FL stage) and Quercetin (the largest accumulation in F stage) under the action of *F3H* and *FLS*. (2.3.1.2.2) Eriodictyol was converted to Luteolin (the largest accumulation in FB stage) by *FNS1*, and then converted to Dihydromyricetin (the largest accumulation in FL stage) by *CYP75A*, *FNS1*, and *F3H*, opening two pathways (2.3.1.2.2.1–2.3.1.2.2.2). (2.3.1.2.2.1) Dihydromyricetin converted to Myricetin (the largest accumulation in W stage). (2.3.1.2.2.2) Dihydromyricetin was converted to Leucodelphinidin, opening two pathways (2.3.1.2.2.2.1–2.3.1.2.2.2.2). (2.3.1.2.2.2.1) Leucodelphinidin was converted to Gollocatechin (the largest accumulation in FL stage) under the action of *ANR*. (2.3.1.2.2.2.2) Leucodelphinidin was converted to Epigallocatechin (the largest accumulation in FL stage) under the action of *ANS* and *ANR*. (2.3.2) Caffeoyl-CoA was converted to 4,2′,4,6′-Tetrahdroxy-3-methoxychalone catalyzed by *CCOAMT* and *CHS*. (2.4) p-Coumaroyl-CoA was converted to Liquiritigenin under the action of *CHS* and *CHI*, opening two pathways (2.4.1–2.4.2). (2.4.1) Liquiritigenin was converted to 6,7,4′-Trihydroxy-flavanone under the action of *CYP71D9*. (2.4.2) Liquiritigenin was converted to 2,7,4′-Trihydroxy-isoflavanone under the action of *CYP93C*, and four pathways were opened at the same time (2.4.2.1–2.4.2.4). (2.4.2.1) 2,7,4′-Trihydroxy-isoflavanone converted to 3,9-Dihydroxypterocarpen. (2.4.2.2) 2,7,4′-Trihydroxy-isoflavanone was converted to Isoformononetin (the largest accumulation in MF stage) under the action of *7-IOMT*. (2.4.2.3) 2,7,4′-Trihydroxy-isoflavanone was converted to Formononetin (the largest accumulation in MF stage) under the action of *HI4OMT* and *HIDH*, opening three pathways (2.4.2.3.1–2.4.2.3.3). (2.4.2.3.1) Formononetin was converted to Medicarpin-3-O-glucoside-6′-malonate (the largest accumulation in MF stage) under the action of *I2′H*, *IFR*, *VR*, *PTS*, *IF7GT*, and *IF7MAT*. (2.4.2.3.2) Formononetin was converted to Formononetin 7-O-glucoside (the largest accumulation in MF stage) under the action of *IF7GT*, and then converted to Formononetin 7-O-glucoside-6′′ -O-malonate (the largest accumulation in MF stage) under the action of *IF7MAT*. (2.4.2.3.3) Formononetin was converted to Calycosin (the largest accumulation in MF stage) under the action of *CYP81E9*, and then finally converted to Maackiain-3-O-glucoside (the largest accumulation in W stage) under the action of *I2*′*H*, *IFR*, and *IF7MAT*. (2.4.2.4) 2,7,4′-Trihydroxy-isoflavanone was converted to Daidzein under the action of HI4OMT and *HIDH*, opening two pathways (2.4.2.4.1–2.4.2.4.2). (2.4.2.4.1) Daidzein was converted to Daidzein 7-O-glucoside (the largest accumulation in FB stage) under the action of *IF7GT*, and then converted to Daidzein 7-O-glucoside-6′′-O-malonate (the largest accumulation in W stage) under the action of *IF7MAT*. (2.4.2.4.2) Daidzein was converted to Formononetin, opening three pathways (the same as 2.4.2.3.1–2.4.2.3.3). In the flavonoid biosynthesis pathway of *A. membranaceus* stems and leaves, Medicarpin and Medicarpin-3-O-glucoside, Medicarpin-3-O-glucoside and Medicarpin-3-O-glucoside-6′-malonate, Formononetin and Formononetin 7-O-glucoside, Formononetin 7-O-glucoside and Formononetin 7-O-glucoside-6′′-O-malonate, Formononetin and Calycosin, and Maackiain and Maackiain-3-O-glucoside achieved two-way conversion. The results provided a strong research basis for further exploration of flavonoid metabolism in *A. membranaceus* stems and leaves.

### 3.8. Screening of Key Genes for Isoquercitrin Biosynthesis

**Figure 8:**
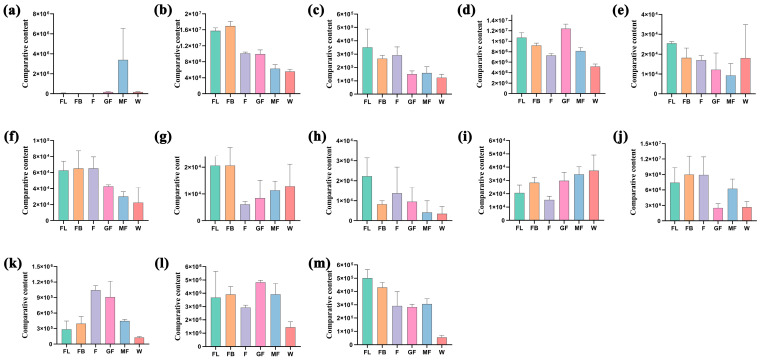
Relative content of differentially accumulated flavonoids in flavonoid and flavonol pathways. (**a**) Acacetin; (**b**) Baimaside; (**c**) Cosmosiin; (**d**) Isoquercitrin; (**e**) Kaempferol; (**f**) Kaempferol-3-O-sophorotrioside; (**g**) Luteolin; (**h**) Luteolin-7-O-glucuronide; (**i**) Myricetin; (**j**) Nicotiflorin; (**k**) Quercetin; (**l**) Quercetin-3-O-sambubioside; and (**m**) Rutin.

Our previous studies have shown that isoquercitrin contained in *A. membranaceus* stems and leaves has significant antioxidant activity. Therefore, this study intends to screen key enzyme genes related to isoquercitrin biosynthesis. In flavone and flavonol biosynthesis (ko00944), FL vs. FB, FL vs. F, FL vs. GF, FL vs. MF, FL vs. W, FB vs. F, FB vs. GF, FB vs. MF, FB vs. W, F vs. GF, F vs. MF, F vs. W, GF vs. MF, GF vs. MF, GF vs. MF, and GF vs. W were enriched in 1, 3, 6, 5, 7, 3, 4, 3, 7, 2, 4, 7, 3, 4, and 5 differentially accumulated flavonoids, respectively (Supplement Table S7). The accumulation of 13 differential flavonoids in the synthesis pathway of flavone and flavonols in different stages was shown in Figure 8. The accumulation of cosmosiin, kaempferol, luteolin-7-O-glucuronide, and rutin was the highest in the FL stage; the accumulation of baimaside, kaempferol-3-O-sophorotrioside, luteolin, and nicotiflorin was the highest in the FB stage; the accumulation of quercetin was the highest in the F stage; the accumulation of isoquercitrin and quercetin-3-O-sambubioside was the highest in the GF stage; the accumulation of acacetin and myricetin was the highest in the MF and W stages. Pearson correlation analysis was performed on 13 DEMs and 38 DEGs annotated in the flavonoid and flavonol biosynthesis pathways. The key genes related to isoquercitrin were screened with *p*-value ≤ 0.05 as the threshold. The results showed that *IF7MAT*, *FG3*, and *UGT78D2* were closely related to the biosynthesis of isoquercitrin (Table 1).

## 4. Discussion

### 4.1. Application of Transcriptome and Metabolome in Mining Key Genes for Secondary Metabolite Biosynthesis

Metabolites are the material basis for plants to exert their active effects. The analysis of the biosynthetic pathways of important secondary metabolites and identification of key enzyme genes in the synthetic pathway have become the main contents of the plant’s functional genomics. The combined analysis of transcriptome and metabolome can effectively identify the key genes involved in the biosynthesis pathway of plant secondary metabolites. In the study of *sinopoodophyllum hexandrum*, 10 key genes in the podophyllotoxin synthesis pathway were identified and synthesized in vitro [39]. In *Garland lily*, multiple genes involved in colchicine synthesis were screened out [40]. In *Glycyrrhiza urulensis*, specific expression genes related to isoflavone and glycyrrhizic acid biosynthesis were found [41]. Multi-omics combined research has further promoted the analysis of key enzymes in the biosynthetic pathway of plant active ingredients such as *Scutellaria baicalensis* and in vitro production [42]. These findings collectively demonstrate that the combination of transcriptomics and metabolomics is a powerful strategy for unraveling the genetic basis of secondary metabolite biosynthesis in plants. In this study, stems and leaves were collected at six stages during the growth and development of *A. membranaceus* for transcriptome sequencing and metabolomics analysis. A total of 184,121 unigenes were obtained by assembling the transcriptome sequencing results, including 52,568 DEGs. Based on the flavonoid metabolic pathway, the expression pattern and KEGG enrichment analysis of DEGs were performed. The results showed that 35 genes involved in flavonoid metabolism in *A. membranaceus* stems and leaves were annotated. The WGCNA and KEGG enrichment analysis of DEGs and DAMs at different growth stages of *Tilia miqueliana* Maxim. leaves underscored the link between gene expression and flavonoid levels [43]. In this study, WGCNA analysis of unigenes obtained by transcriptome sequencing showed that *PAL*, *CHI*, *AMIE*, *CAD*, and *PRX* played a core regulatory role in flavonoid biosynthesis and were hub genes of flavonoid biosynthesis during the development of *A. membranaceus* stems and leaves. A total of 944 metabolites were identified by metabolomics analysis, of which flavonoids were the most, accounting for 39.51%.

### 4.2. Construction of Metabolic Regulation Network of Flavonoids in A. membranaceus Stems and Leaves

Flavonoids, as one of the three major secondary metabolites of plants, can not only regulate their own growth and development but also help resist the adverse external environment. The molecular mechanism of the synthesis and regulation of flavonoids has been a hot topic, and the research reports on the molecular regulation of flavonoids have gradually increased in recent years. The study of flavonoids in *Carthamus tinctorius* L. showed that the high expression of the *CHS1* gene increased the content of quinone chalcone glycosides by 20–30% and decreased the accumulation of quercetin-3-β-D-glucoside and quercetin by 48% and 63%, respectively [44]. The study of Artemisia annua L. and *Ginkgo biloba* L. showed that *CHS* and *CHI* genes were significantly correlated with the accumulation of flavonoids [45,46]. In addition, the recombinant fusion protein of the flavonol synthase gene *FLS* in Chrysanthemum morifolium Ramat. can catalyze dihydroquercetin to produce quercetin [47]. Transcriptome and metabolome analysis on *Cinnamomum camphora* have identified *Cc4CL_1* and *Cc4CL_9* acting as robust positive regulators of rutin biosynthesis, contrasting sharply with the strong inhibitory role of *CcC4H_1* [48]. At present, the research on flavonoids in *A. membranaceus* mainly focuses on the biological activity of active compounds, and there are relatively few studies on its biosynthesis, especially stems and leaves. A continuous flavonoid synthesis pathway of ‘Zizhouhuangqi’ was obtained by using transcriptome and metabolome data, and 15 secondary metabolites and 16 enzymes that catalyze the synthesis of flavonoids were annotated [49]. In this study, high-throughput sequencing technology was used to perform transcriptome sequencing and metabolomics analysis at different growth and development stages of A. *membranaceus* stems and leaves. A total of 32 differentially accumulated flavonoids and 109 differentially accumulated unigenes were annotated in the flavonoid metabolic pathway. At the same time, they were combined and analyzed to construct the flavonoid metabolic regulatory network starting from phenylalanine during the growth and development of *A. membranaceus* stems and leaves. A branch of the biosynthesis of Calycosin and Biochanin A isoflavones was found downstream of the production of coumaroyl-CoA, which was consistent with the research of Wu et al. [50]. In addition to the Coumaroyl-CoA branch in the downstream pathway of Cinnamoyl-CoA, this study also found the Pinocembrin chalcone branch, which was completed by Pinobanksim 3-acetate. As a downstream pathway of Formononetin, Calycosin, and Formononetin branches have been reported in previous studies [50,51], but this study found another branch, which is completed with Medicarpin-3-O-glucoside-6′-malonate. In the upstream pathway of Formononetin, Daidzein can be further acylated to form Daidzein 7-O-glucoside-6″-O-malonate, confirming the activity of *IF7GT* and *IF7MAT*. Interestingly, this study identified Myricetin, Gallocatechin, and Epigallocatechin in the downstream pathway of Luteolin, while Wu et al. found Chrysoerial and its derivatives [50]. It should be noted that in transcriptome analysis, multiple unigenes may be annotated as the same enzyme, mainly because these unigenes belong to different alternatively spliced transcripts and specific gene families [52]. In this study, multiple genes encoding the same enzyme were also identified in the *A. membranaceus* stems and leaves (Figure 8).

### 4.3. Biological Activity of Isoquercetin and Excavation of Key Genes for Its Biosynthesis

Isoquercitrin is a flavonol with a molecule of glucose attached to the 3-position of the basic nucleus of flavone. It is almost insoluble in water and easily soluble in organic solvents such as methanol and ethanol [53]. Isoquercitrin has good antioxidant activity and can effectively scavenge DPPH free radicals, superoxide anions, hydroxyl radicals, and nitrite [54]. Isoquercitrin can reduce the oxidative damage of retinal ganglion caused by hydrogen peroxide and improve the tolerance of Saccharomyces cerevisiae to hydrogen peroxide and menadione [55,56]. Isoquercitrin has a protective effect on oxidative damage induced by H_2_O_2_ and tert-butyl hydroperoxide, and can reduce Cd^2+^-induced liver and kidney toxicity [57,58]. In addition, it also exhibits a variety of beneficial activities, such as anti-tumor, anti-bacterial, anti-viral, anti-hypertensive, lipid-lowering, anti-atherosclerosis, hypoglycemic, anti-osteoporosis, and neuroprotection [59,60,61,62,63,64,65,66,67].

Our previous studies have shown that isoquercitrin contained in *A. membranaceus* stems and leaves has significant antioxidant activity [29]. Wang et al. also isolated the flavonoids from *A. membranaceus* leaves by preparative high-performance liquid chromatography [68]. In this study, the transcriptome and metabolome of 18 samples from six stages were sequenced and analyzed. The results showed that three genes that may be related to isoquercitrin biosynthesis were screened, namely *IF7MAT*, *FG3*, and *UGT78D2*. In plants, flavonoids exist in a variety of modified forms such as hydroxylation, methylation, acylation, and glycosylation, among which glycosylated flavonoids are the most common natural compounds. Glycosylation is mainly catalyzed by glycosyltransferases, and flavonoid C-glycosides and O-glycosides are both catalyzed by uridine diphosphate-sugar-dependent glycosyltransferases (UGTs) [69]. Xu et al. research showed that the decrease of *IF7MAT* expression after hydrogen treatment of germinated soybean under UV-B radiation promoted the conversion of soybean isoflavone glycosides to aglycones [70]. Wu’s study also identified the expression of the *IF7MAT* gene in *A. membranaceus* stems and leaves [50]. Glycosylation is often the final step in flavonoid biosynthesis and is catalyzed by the family 1 glycosyltransferases referred to as UDP glycosyltransferases (UGTs) that transfer a glycosyl moiety from UDP sugars to a wide range of acceptors including flavonoids [71]. Flavonoid glycoside glycosyltransferases (FGGs) attach additional sugars to an existing sugar moiety of flavonol glycosides (FGs), resulting in a wide variety of binding positions and sugar combinations [72]. The function of these key genes will be identified in our future work.

## 5. Conclusions

In this study, widely targeted metabolomics and transcriptomics techniques were used to analyze the six different growth stages of *A. membranaceus* stems and leaves. A total of 52,568 DEGs and 944 DAMs were identified. The WGCNA analysis of unigenes identified five hub genes, namely *PAL*, *CHI*, *AMIE*, *CAD*, and *PRX*, which play a core regulatory role in flavonoid biosynthesis in *A. membranaceus* stems and leaves. At the same time, the combined analysis of transcriptomics and metabolomics constructed a flavonoid metabolic regulatory network during the growth and development of *A. membranaceus* stems and leaves. Three genes, *IF7MAT*, *FG3*, and *UGT78D2*, which may be related to isoquercitrin biosynthesis, were screened. The results provide a theoretical basis for the biosynthesis and molecular breeding of flavonoids in *A. membranaceus*.

## Figures and Tables

**Table 1:** Key genes of isoquercitrin biosynthesis.

Abbreviations	Name	ID	*p*-Value
*FG3*	Flavonol-3-O-glucoside/galactoside glucosyltransferase	Cluster-72196.0	0.001142266
		Cluster-28836.6	0.017451255
		Cluster-72196.7	0.032142939
		Cluster-28836.10	0.041314142
*UGT78D2*	Flavonol 3-O-glucosyltransferase	Cluster-36532.0	0.03244486
*IF7MAT*	Isoflavone 7-O-glucoside-6′′-O-malonyltransferase	Cluster-49597.0	0.000126991
		Cluster-61056.0	0.002868794
		Cluster-62165.3	0.003190675
		Cluster-61377.0	0.005416846
		Cluster-62165.2	0.007126815
		Cluster-50242.19	0.009885351
		Cluster-50242.3	0.010056192
		Cluster-68275.0	0.010825818
		Cluster-62165.4	0.01796377
		Cluster-50242.12	0.019924075
		Cluster-50242.10	0.048326017

## Data Availability

The datasets generated and/or analyzed during the current study are available in the National Center for Biotechnology Information repository, [ACCESSION NUMBER: PRJNA1182064, https://dataview.ncbi.nlm.nih.gov/object/PRJNA1182064?reviewer=ka3q61l14ncsa9t4v2u3pn7rf (accessed on 2 November 2025)].

## Supplementary Materials

The following supporting information can be downloaded at: https://www.mdpi.com/article/10.3390/foods15020218/s1, Figure S1: Transcriptome analysis at different developmental stages; Figure S2: TIC analysis of mixed sample mass spectrum, MRM detection of metabolites, and integral correction of quantitative analysis of some metabolites; Figure S3: The CV value distribution map, clustering heat-map, correlation diagram, PCA plot, and metabolite class composition in this study; Figure S4: OPLS-DA analysis of different groups of *A. membranaceus* stems and leaves; Figure S5: OPLS-DA model validation; Table S1: Transcriptome sequencing results of *A. membranaceus* stems and leaves; Table S2: The transcriptome data of *A. membranous* stems and leaves during the six development stages; Table S3: Genes involved in flavonoid synthesis during fruit development of *A. membranaceus*; Table S4: Statistical table of gene modules in *A. membranaceus* stem and leaf samples; Table S5: Gene Set in four modules that participate in flavonoid biosynthesis; Table S6: Differentially accumulated flavonoid compounds in 15 comparison groups; Table S7: Differentially accumulated flavonoid compounds in flavone and flavonol biosynthesis.

## Abbreviations

The following abbreviations are used in this manuscript:

*4CL*	4-coumarate--CoA ligase
*7-IOMT*	isoflavone-7-O-methyltransferase
*AAT*	aspartate-prephenate aminotransferase
*AMIE*	amidase
*ANR*	anthocyanidin reductase
*ANS*	anthocyanidin synthase
*AOC3*	primary-amine oxidase
*CAD*	cinnamyl-alcohol dehydrogenase
CC	Cellular component
*CCOAMT*	caffeoyl-CoA O-methyltransferase
*CCR*	cinnamoyl-CoA reductase
CDS	Coding sequence
*CHI*	chalcone isomerase
*CHR*	chalcone reductase
*CHS*	chalcone synthase
*COMT*	caffeic acid 3-O-methyltransferase
*CSE*	caffeoylshikimate esterase
CV	Coefficient of variation
*CYP450*	cytochromeP450
*CYP71D9*	flavonoid 6-hydroxylase
*CYP73A*	trans-cinnamate 4-monooxygenase
*CYP75A*	flavonoid 3′,5′-hydroxylase
*CYP81E9*	isoflavone 3′-hydroxylase
*CYP93C*	2-hydroxyisoflavanone synthase
*CYP98A*	5-O-(4-coumaroyl)-D-quinate 3′-monooxygenase
DAMs	Differentially accumulated metabolites
*DDC*	aromatic-L-amino-acid
DEGs	Differentially expressed genes
*DFR*	flavanone 4-reductase
DPPH	1,1-Diphenyl-2-picrylhydrazyl radical 2,2-Diphenyl-1-(2,4,6-trinitrophenyl)hydrazyl
ECDF	Empirical cumulative distribution function
F	Flowering stage
*F3H*	naringenin 3-dioxygenase
*F5H*	ferulate-5-hydroxylase
FB	Flower bud stage
FDR	False discovery rate
FL	Flowerless stage
*FLS*	flavonol synthase
*FNS1*	flavone synthase I
*FG3*	flavonol-3-O-glucoside/galactoside glucosyltransferase
FPKM	Fragments Per Kilobase of transcript per Million fragments mapped
GF	Green fruit stage
GO	Gene Ontology
HCA	Hierarchical cluster analysis
*HCT*	shikimate O-hydroxycinnamoyltransferase
*HI4OMT*	isoflavone 4′-O-methyltransferase
*HIDH*	2-hydroxyisoflavanone dehydratase
*HPPD*	4-hydroxyphenylpyruvate dioxygenase
*HPPR*	hydroxyphenylpyruvate reductase
*I2′H*	isoflavone/4′-methoxyisoflavone 2′-hydroxylase
*IF7GT*	isoflavone 7-O-glucosyltransferase
*IF7MAT*	isoflavone 7-O-glucoside-6″-O-malonyltransferase
*IFR*	isoflavone reductase
KEGG	Kyoto Encyclopedia of Genes and Genomes
KOG	EuKaryotic Orthologous Groups
MF	Mature fruit stage
MF	Molecular function
*MIF*	phenylpyruvate tautomerase
MRM	Multiple reaction monitoring
MSEA	Metabolite set enrichment analysis
NR	Non-Redundant
OPLS-DA	Orthogonal projections to latent structures-discriminant analysis
*PAL*	phenylalanine ammonia-lyase
PCA	Principal component analysis
*PDH*	phenylalanine dehydrogenas
*PGT1*	phlorizin synthase
*PRX*	peroxidase
*PTR*	pterocarpan reductase
*PTS*	pterocarpan synthase
QC	Quality control
*REF1*	coniferyl-aldehyde dehydrogenase
*TAT*	tyrosine aminotransferase
TF	Transcription factor
TIC	Total ion current
BP	Biological process
TrEMBL	Translation of EMBL
*UGT*s	Uridine diphosphate-sugar-dependent glycosyltransferases
*UGT78D2*	flavonol 3-O-glucosyltransferase
VIP	Variable importance in projection
*VR*	vestitone reductase
W	Withering stage
WGCNA	weighted gene co-expression network analysis
