# Supplementary material for: Metabolomics and Transcriptomics Analyses Explore the Genes Related to the Biosynthesis of Antioxidant Active Ingredient Isoquercetin
Source: Foods. 2026 Jan 8;15(2):218. doi: 10.3390/foods15020218 (PMC12839654; doi:10.3390/foods15020218)
Supplement: Supplementary file 1 [file foods-15-00218-s001.zip › Figure S1.pdf]

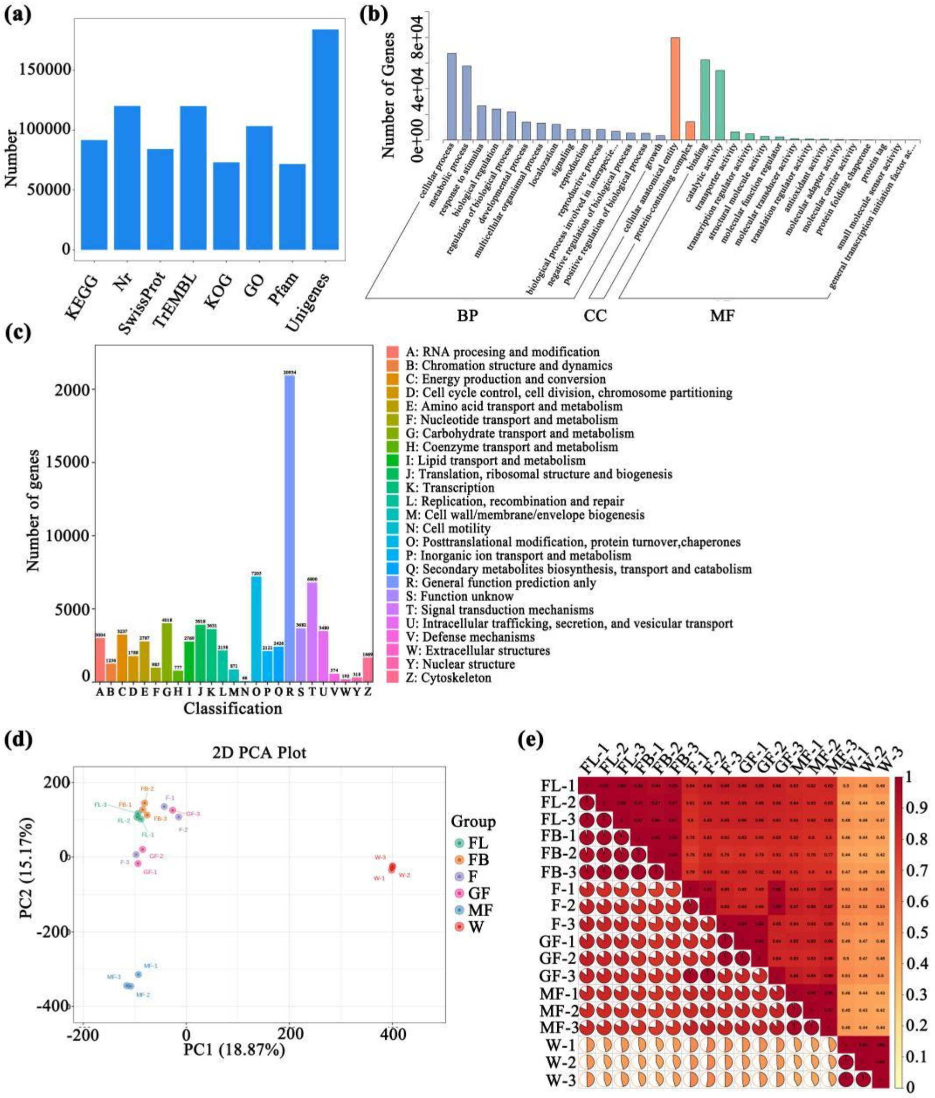

Supplement Figure S1. Transcriptome analysis at different developmental stages.

(a): Unigene annotation; (b): GO database annotation; (c): KOG database annotation; (d): PCA analysis; (e): Correlation diagram of samples.
