# Supplementary material for: Metabolomics and Transcriptomics Analyses Explore the Genes Related to the Biosynthesis of Antioxidant Active Ingredient Isoquercetin
Source: Foods. 2026 Jan 8;15(2):218. doi: 10.3390/foods15020218 (PMC12839654; doi:10.3390/foods15020218)
Supplement: Supplementary file 1 [file foods-15-00218-s001.zip › Figure S3.pdf]

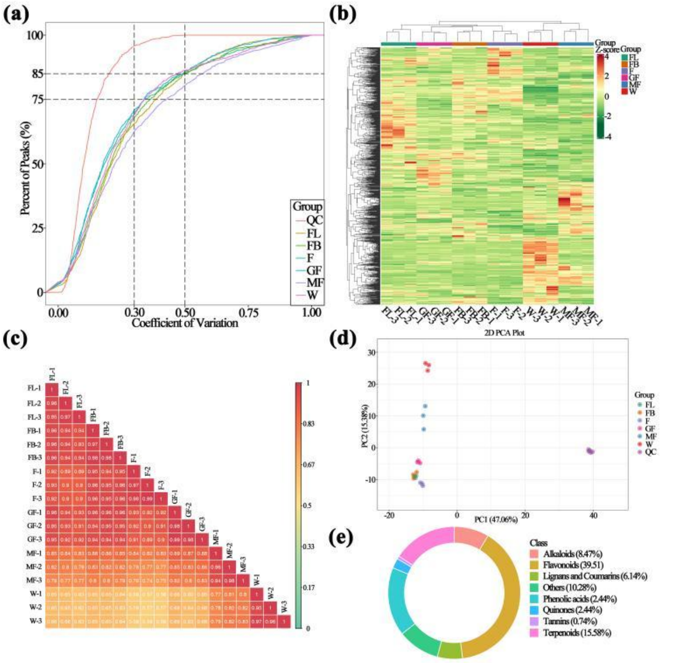

Supplement Figure S3. The CV value distribution map, clustering heat-map, correlation diagram, PCA plot, and metabolite class composition in this study.

(a): CV distribution; The CV value distribution map for all samples, with the horizontal axis representing the CV value and the vertical axis representing the proportion of the number of substances less than the corresponding CV value to the total number of substances. Different colors represented different grouped samples, and QC represented quality control samples. (b): The cluster heatmap of the overall samples of each group. (c): Correlation analysis of the overall samples of each group. (d): The PCA plot for each group of samples; (e): The circular plot of overall metabolite class composition, with values in parentheses indicating the percentage of each categorical metabolite to the total metabolite.
