# Supplementary material for: Metabolomics and Transcriptomics Analyses Explore the Genes Related to the Biosynthesis of Antioxidant Active Ingredient Isoquercetin
Source: Foods. 2026 Jan 8;15(2):218. doi: 10.3390/foods15020218 (PMC12839654; doi:10.3390/foods15020218)
Supplement: Supplementary file 1 [file foods-15-00218-s001.zip › Figure S5.pdf]

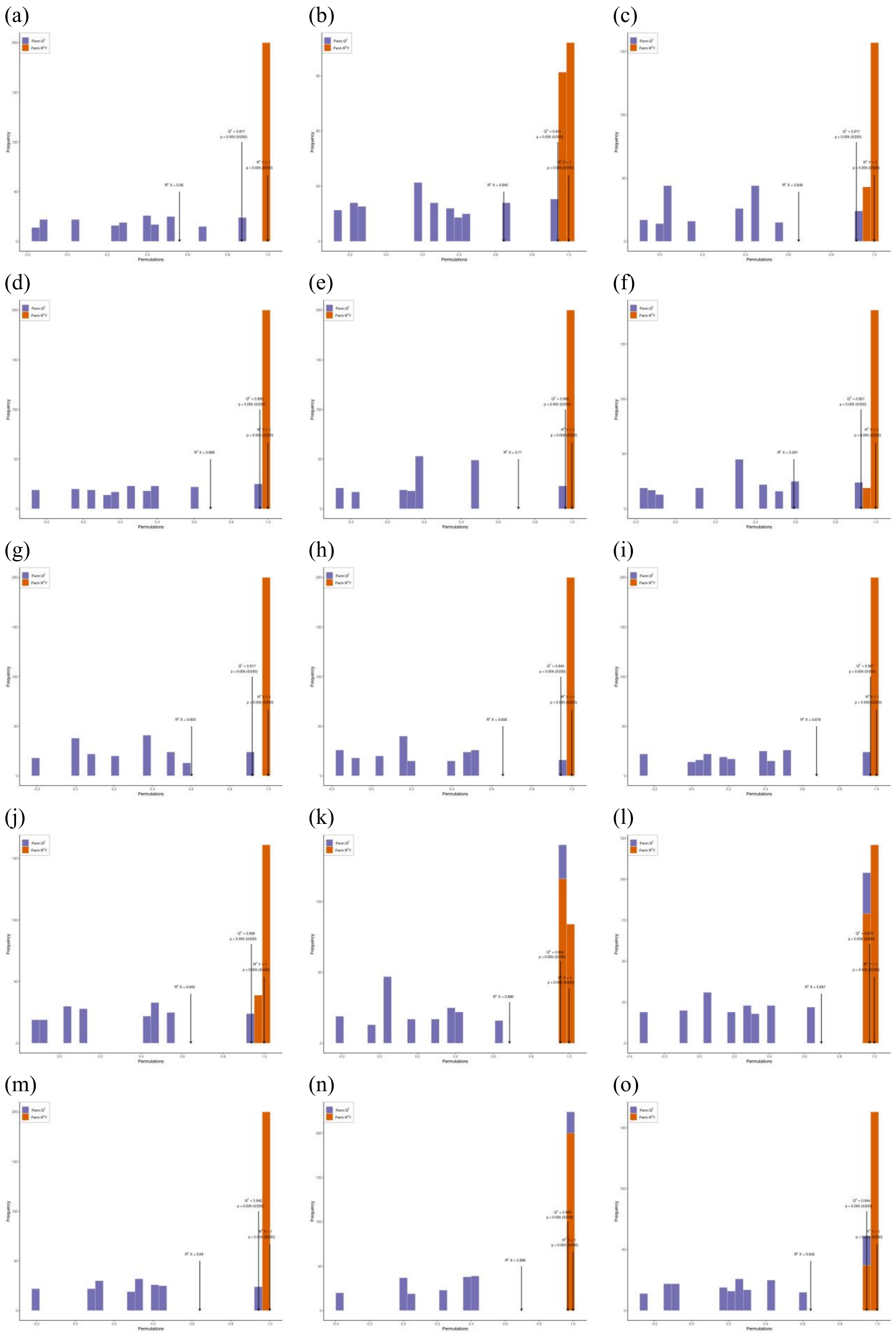

Supplement Figure S5. OPLS-DA model validation

(a): FL vs. FB; (b): FL vs. F; (c): FL vs. GF; (d): FL vs. MF; (e): FL vs. W; (f): FB vs. F; (g): F vs. GF; (h): FB vs. MF; (i): FB vs. W; (j): F vs. GF; (k): F vs. MF; (l): F vs. W; (m): GF vs. MF; (n): GF vs. W; (o): MF vs. W. The horizontal axis represented the R^2Y and Q^2

values of the model, while the vertical axis represented the frequency of model classification effects in 200 randomly arranged combination experiments. In the figure, orange represented the random grouping model R^2Y, purple represented the random grouping model Q^2, and the black arrow represented the R^2X, R^2Y, and Q^2 values of the original model.
