# Supplementary material for: Metabolomics and Transcriptomics Analyses Explore the Genes Related to the Biosynthesis of Antioxidant Active Ingredient Isoquercetin
Source: Foods. 2026 Jan 8;15(2):218. doi: 10.3390/foods15020218 (PMC12839654; doi:10.3390/foods15020218)
Supplement: Supplementary file 1 [file foods-15-00218-s001.zip › Table S1.pdf]

Table S1 Transcriptome sequencing results of *A. membranaceus* stems and leaves.

Sample	Raw Reads	Clean Reads	Clean Base（G）	Error Rate（%）	Q30（%）	GC Content（%）
FL-1	112840140	101845364	15.28	0.03	93.27	42.54
FL-2	82037576	73442486	11.02	0.03	92.57	42.69
FL-3	71998008	67087626	10.06	0.04	91.95	42.5
FB-1	116352308	104322458	15.65	0.03	93.17	42.5
FB-2	84596948	77397482	11.61	0.03	92.47	42.51
FB-3	82894908	69358718	10.4	0.03	93.07	42.86
F-1	97933274	90828534	13.62	0.03	92.88	42.6
F-2	92341336	79734670	11.96	0.03	92.83	42.76
F-3	94084744	83797774	12.57	0.03	92.61	42.6
GF-1	92470388	84496436	12.67	0.03	93.39	42.61
GF-2	64891250	61834192	9.28	0.04	91.1	42.55
GF-3	85048734	77561468	11.63	0.04	92.26	42.69
MF-1	116987322	100876802	15.13	0.03	93.37	42.65
MF-2	73636552	68280522	10.24	0.04	92.09	42.66
MF-3	74003796	69207196	10.38	0.04	92.1	42.8
W-1	80205960	73997916	11.1	0.03	93.16	42.4
W-2	81767766	70908128	10.64	0.03	93.05	42.5
W-3	98538070	81882380	12.28	0.03	92.68	42.75
