# Supplementary material for: Metabolomics and Transcriptomics Analyses Explore the Genes Related to the Biosynthesis of Antioxidant Active Ingredient Isoquercetin
Source: Foods. 2026 Jan 8;15(2):218. doi: 10.3390/foods15020218 (PMC12839654; doi:10.3390/foods15020218)
Supplement: Supplementary file 1 [file foods-15-00218-s001.zip › Table S3.pdf]

Table S3 Genes involved in flavonoid synthesis during fruit development of *A. membranaceus*

Abbreviations	Name
4CL	4-coumarate--CoA ligase
7-IOMT	isoflavone-7-O-methyltransferase
AMIE	amidase
ANR	anthocyanidin reductase
AOC3	primary-amine oxidase
CAD	cinnamyl-alcohol dehydrogenase
CCOAMT	caffeoyl-CoA O-methyltransferase
CCR	cinnamoyl-CoA reductase
CHI	chalcone isomerase
CHS	chalcone synthase
COMT	caffeic acid 3-O-methyltransferase
CSE	caffeoylshikimate esterase
CYP71D9	flavonoid 6-hydroxylase
CYP98A	5-O-(4-coumaroyl)-D-quinate 3'-monooxygenase
DDC	aromatic-L-amino-acid
DFR	flavanone 4-reductase
F3H	naringenin 3-dioxygenase
F5H	ferulate-5-hydroxylase
FLS	flavonol synthase
GOT1	aspartate aminotransferase
HCT	shikimate O-hydroxycinnamoyltransferase
HI4OMT	isoflavone 4'-O-methyltransferase
HIDH	2-hydroxyisoflavanone dehydratase
HPPD	4-hydroxyphenylpyruvate dioxygenase
HPPR	hydroxyphenylpyruvate reductase
I2'H	isoflavone/4'-methoxyisoflavone 2'-hydroxylase
IF7MAT	isoflavone 7-O-glucoside-6"-O-malonyltransferase
MIF	phenylpyruvate tautomerase
PAL	phenylalanine ammonia-lyase
PGT1	phlorizin synthase
PRX	peroxidase
PTR	pterocarpan reductase
PTS	pterocarpan synthase
REF1	coniferyl-aldehyde dehydrogenase
TAT	tyrosine aminotransferase
VR	vestitone reductase
