# Supplementary material for: Metabolomics and Transcriptomics Analyses Explore the Genes Related to the Biosynthesis of Antioxidant Active Ingredient Isoquercetin
Source: Foods. 2026 Jan 8;15(2):218. doi: 10.3390/foods15020218 (PMC12839654; doi:10.3390/foods15020218)
Supplement: Supplementary file 1 [file foods-15-00218-s001.zip › Table S4.pdf]

Table S4 Statistical table of gene modules in *A. membranaceus* stem and leaf samples

Module	Number	Module	Number	Module	Number
turquoise	3995	tan	1641	darkturquoise	280
blue	3895	salmon	1469	darkgrey	229
brown	2711	cyan	1158	orange	158
yellow	2607	midnightblue	1033	darkorange	157
green	2052	lightcyan	641	white	143
red	1980	grey60	607	skyblue	121
black	1923	lightgreen	530	saddlebrown	118
pink	1849	lightyellow	460	paleturquoise	70
magenta	1777	royalblue	439	steelblue	70
purple	1759	darkred	400	violet	61
greenyellow	1656	darkgreen	323		
