# Supplementary material for: Metabolomics and Transcriptomics Analyses Explore the Genes Related to the Biosynthesis of Antioxidant Active Ingredient Isoquercetin
Source: Foods. 2026 Jan 8;15(2):218. doi: 10.3390/foods15020218 (PMC12839654; doi:10.3390/foods15020218)
Supplement: Supplementary file 1 [file foods-15-00218-s001.zip › Table S5.pdf]

Table S5 Gene Set in four modules that participate in flavonoid biosynthesis

Gene Set	Gene
black	*4CL; 7-IOMT; AAT; AMIE; ANR; AOC3; CAD; CCOAMT; CCR; CHI; CHR; COMT; CSE; CYP71D9; CYP73A; CYP98A; DDC; F3H; F5H; FLS; HCT; HIDH; IF7MAT; IFR; MIF; PAL; PDH; PGT1; PRX; PTS; TAT; VR*
cyan	*AMIE; MIF*
midnightblue	*CAD; CCR; COMT; CSE; PAL; PRX*
steelblue	*PAL; PRX*
