# Supplementary figures and images for: Metabolomics and Transcriptomics Analyses Explore the Genes Related to the Biosynthesis of Antioxidant Active Ingredient Isoquercetin
Source: Foods. 2026 Jan 8;15(2):218. doi: 10.3390/foods15020218 (PMC12839654; doi:10.3390/foods15020218)

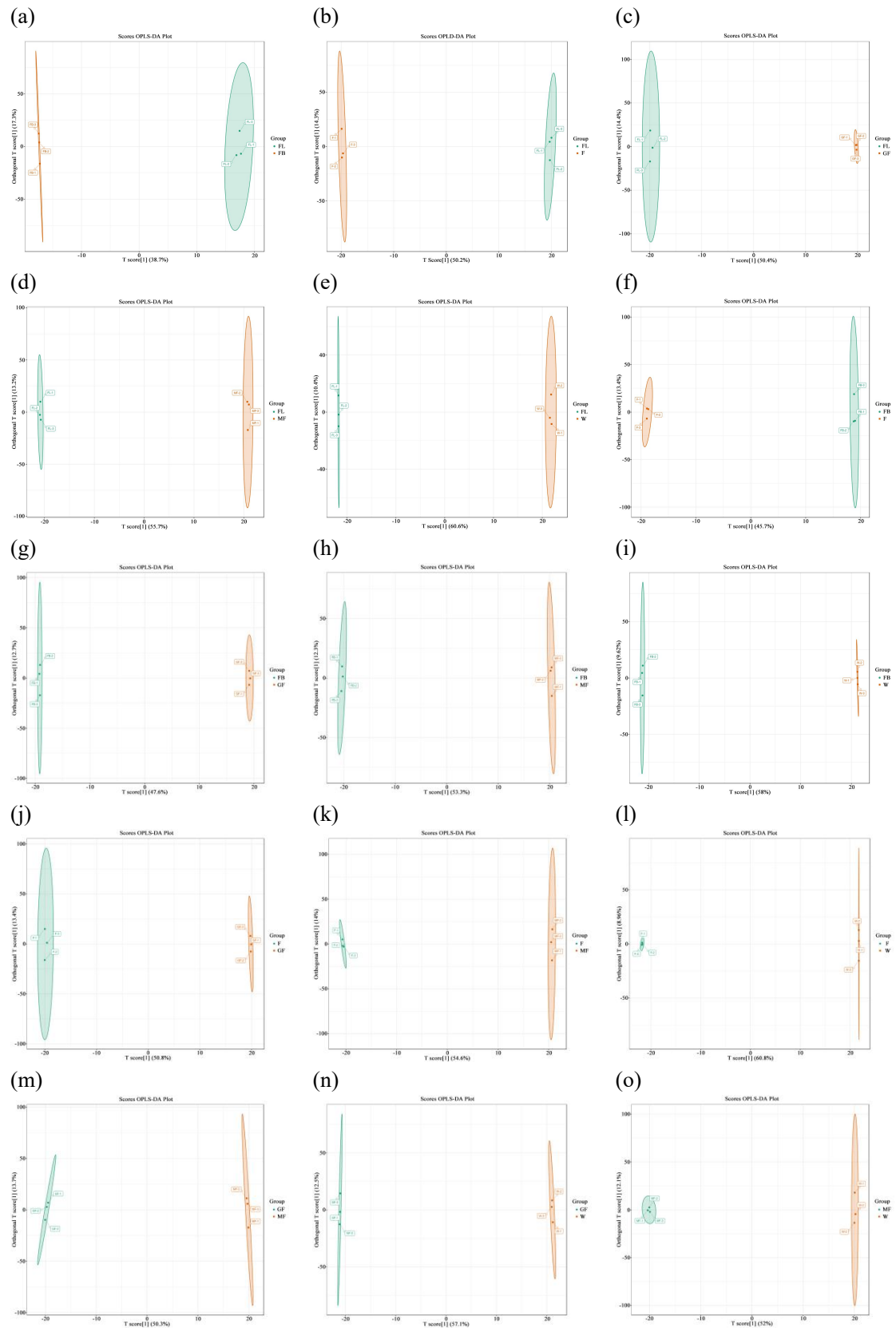

Supplement: Supplementary file 1 [file foods-15-00218-s001.zip › Figure S4.pdf]
