# Supplementary material for: Metabolomics and Transcriptomics Analyses Explore the Genes Related to the Biosynthesis of Antioxidant Active Ingredient Isoquercetin
Source: Foods. 2026 Jan 8;15(2):218. doi: 10.3390/foods15020218 (PMC12839654; doi:10.3390/foods15020218)
Supplement: Supplementary file 1 [file Figure S2.pdf › Figure S2.pdf]

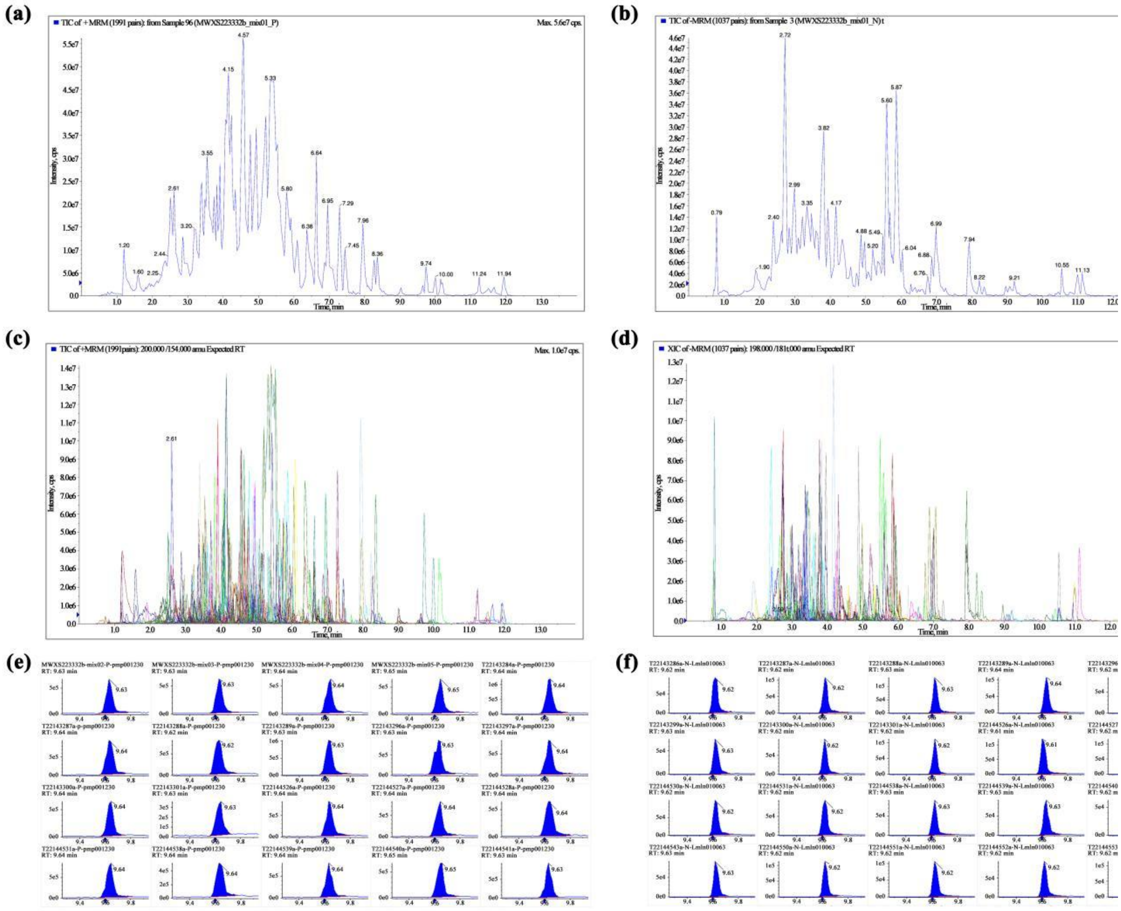

Supplement Figure S2. TIC analysis of mixed sample mass spectrum, MRM detection of metabolites, and integral correction of quantitative analysis of some metabolites.

(a): TIC positive ion mode; (b): TIC negative ion mode; (c): MRM positive ion mode; (d): MRM negative ion mode; (e): positive ion mode integral correction; and (f): negative ion mode integral correction.
